# Sudden Blindness

**Published:** 1909-01-09

**Authors:** 


### Ophthalmology.

### SUDDEN BLINDNESS.

The causes of sudden loss of vision, either in one eye or both, may be roughly divided into two groups. The first includes changes in the eye itself, the optic nerve, and the orbital cavity. The second, constitutional derangements which interfere permanently or temporarily with the function of the eye as a sense organ. Those changes included in the first group account for by far the greatest number of such cases. In a large number the blindness is happily only temporary, though it may at first be complete. Many of the changes in the eye itself are local manifestations of general constitutional disturbance, and, though primarily belonging to the second group, merge into the first.

A patient may become suddenly blind while under treatment for some disease where sudden loss of vision is a possibility or even a probability, as in valvular heart disease and renal disease; or when he is in apparently good health. Blindness occurring in one eye only is much more frequent than the bilateral condition, and may usually be looked upon as caused by changes within the eye itself.

In the first group hæmorrhage into the vitreous is a common cause, and it may recur at intervals, as occasionally happens in young men between the ages of 20 to 35. Hæmophilics also, though rarely, suffer from this. In females such hæmorrhages have been known to occur at or about the menstrual periods, especially in cases of sudden suppression. Hæmorrhages of quite moderate size may occur in the neighbourhood of the macula, and, provided they are sufficiently extensive to cover the macula itself, will cause sudden loss of vision. Peripheral vision may still be found on testing when direct central vision has been thus lost; hand movements on each side, above, and below will then only be seen. These hæmorrhages are from the small veins almost invariably; hæmorrhages from the large venous trunks or from retinal arteries give rise to an increase in tension and to acute pain, as well as to loss of vision.

Detachment of the retina causes sudden loss of vision; but not necessarily by reason of the whole retina becoming detached, since the separation of quite a small portion of it so disturbs the rest that it temporarily loses its function. Vision returns within a few days, but remains greatly diminished, and in one part of the field is absent. Detachment of the retina and hæmorrhage into the vitreous may take place simultaneously, or detachment may be brought about after a hæmorrhage.

Embolism of the central artery of the retina in all probability causes the most rapid loss of vision of any affection. Thrombosis of the central vein gives rise to complete blindness, but the onset is not quite so rapid as in embolism of the artery. Hæmorrhage into the sheath of the optic nerve behind the globe at times has given rise to sudden loss of vision, but is extremely difficult to diagnose.

In myopia of high degree sudden loss of vision usually denotes detachment of the retina. In cases of inflammation of the optic nerve behind the globe (retro-bulbar neuritis) sudden blindness may take place, but it is usually preceded by diminished visual acuity for a day or two. It should always be borne in mind that a patient may only suddenly realise that one eye is blind, though it has been blind for some time, possibly throughout life. In testing vision it is not by any means uncommon to find that one eye has perfect visual acuity, whereas the other has less than one-tenth of the normal, and yet the patient is unaware that he is not able to see with both eyes.

The second group, constitutional derangements, includes cases in which no ophthalmoscopic changes are found, and in which no apparent cause exists for the sudden loss of vision, which is absolute though not permanent. Cases of this nature occur in patients of a neurotic temperament, and in those suffering from arterio-sclerosis. Constriction of the retinal vessels or of the central artery before its entrance into the nerve, or in the nerve itself, is in all probability the actual cause; no apparent changes in the retinal vessels are noticeable with the ophthalmoscope during the attack, which as a rule lasts but for a few minutes. Slight pressure applied to the normal eyeball continuously for from half to three-quarters of a minute will bring about blindness in it to the extent of inability to see a naked electric light, but ophthalmoscopic examination while the eye remains thus artificially blinded reveals no change in either the vessels or retina; the vision returns, as a rule, rapidly, on release of the pressure. In this case the pressure interferes with the normal supply of blood to the retina, which immediately begins to lose its function in consequence. Attacks of loss of vision of this nature occur in uræmia most frequently. Excessive hæmorrhages, gastric, uterine, or traumatic, and very occasionally epistaxis, will cause sudden blindness, but as a rule this does not come on till a day or so after the hæmorrhage, and unless the vessels are diseased gradually passes off.

During scarlet fever the patient may suddenly become blind, but this loss of vision is usually accompanied by extreme headache, convulsions, or cerebral symptoms, and is in all probability uræmic. Severe cases of malaria are occasionally complicated by sudden loss of vision, coming on with the rigor and passing off with the onset of the sweating stage; the pupils during the blindness react normally. Here the cause is either a toxic or vascular constriction, most probably the latter, since it passes off with the general dilatation of the vessels and sweating. Hæmorrhages into the vitreous have also been noticed after such an attack. Patients who suffer from migraine occasionally become blind for some few minutes, the blindness affecting their peripheral vision first, then the central, and clearing off in the same order, the peripheral returning first. Sudden blindness has been met with in patients suffering from rheumatic fever, usually during the worst period of the disease; normal vision is slowly recovered. This has been attributed to impurity of the salicylate of soda, though this explanation is not by any means established. No changes have been observed in the fundus to account for it.

